# Chemoradiotherapy-Induced CD4^+^ and CD8^+^ T-Cell Alterations to Predict Patient Outcomes in Esophageal Squamous Cell Carcinoma

**DOI:** 10.3389/fonc.2019.00073

**Published:** 2019-02-15

**Authors:** Xi Chen, Wencheng Zhang, Dong Qian, Yong Guan, Yuwen Wang, Hualei Zhang, Puchun Er, Cihui Yan, Yueguo Li, Xiubao Ren, Qingsong Pang, Ping Wang

**Affiliations:** ^1^>Department of Radiation Oncology, Tianjin Medical University Cancer Institute and Hospital, National Clinical Research Center for Cancer, Key Laboratory of Cancer Prevention and Therapy, Tianjin, Tianjin's Clinical Research Center for Cancer, Tianjin, China; ^2^Department of Immunology,Tianjin Medical University Cancer Institute and Hospital, National Clinical Research Center for Cancer, Key Laboratory of Cancer Prevention and Therapy, Tianjin, Tianjin's Clinical Research Center for Cancer, Tianjin, China; ^3^Department of Clinical Laboratory, Tianjin Medical University Cancer Institute and Hospital, National Clinical Research Center for Cancer, Key Laboratory of Cancer Prevention and Therapy, Tianjin, Tianjin's Clinical Research Center for Cancer, Tianjin, China

**Keywords:** esophageal squamous cell carcinoma, clinical outcomes, CD4^+^ T-cell, CD8^+^ T-cell, chemoradiotherapy

## Abstract

**Purpose and Objectives:** Chemoradiotherapy (CRT) is an important component of treatment for patients with locally advanced esophageal squamous cell carcinoma (ESCC). Recent research findings support the role of CRT in activating an anti-tumor immune response. However, predictors of CRT efficacy are not fully understood. The aim of this study was to measure CRT-induced changes to lymphocyte subpopulations and to evaluate the prognostic value of lymphocyte alterations for patients with ESCC.

**Materials and Methods:** In total, this pilot study enrolled 64 patients with ESCC who received neo-adjuvant CRT or definitive CRT. Peripheral blood samples were collected before and during treatment and were analyzed by flow cytometry for CD19, CD3, CD4, CD8, CD56, and CD16. Relationships between lymphocyte subset alterations and overall survival (OS) and progression-free survival (PFS) were evaluated using the log-rank test and a Cox regression model.

**Results:** The median follow-up period was 11.8 months (range, 4.0–20.2 months). Compared to pre-treatment specimens, post-treatment blood samples had decreased proportions of CD19^+^ B-cells and increased proportions of CD3^+^ and CD8^+^ T-cells (all *P* < 0.05). Univariate and multivariate analysis showed that increased CD4^+^ T-cell ratios after CRT independently predicted superior PFS (hazard ratio [HR] = 0.383; 95% confidence interval [CI] = 0.173–0.848, *P* = 0.017) and that increased CD8^+^ T-cell ratios predicted improved OS (HR = 0.258; 95% CI = 0.083–0.802, *P* = 0.019). Patients with both increased CD4^+^ and CD8^+^ ratios had a superior PFS and OS, compared to patients with an increased CD4^+^ ratio only or CD8^+^ ratio only or neither (1-year PFS rate 63 vs. 25%, 1-year OS rate 80 vs. 62%, *P* = 0.005 and 0.025, respectively).

**Conclusions:** CRT-induced increases in CD4^+^ and CD8^+^ T-cell ratios are reliable biomarker predictors of survival in patients with ESCC.

## Introduction

Esophageal carcinoma is a leading cause of cancer and cancer death worldwide. Esophageal squamous cell carcinoma (ESCC) is the major form of esophageal carcinoma in Eastern Europe and Asia, with a 5-year overall survival of 15–25% (1–3). In addition to well-described clinical and therapeutic determinants of survival, the tumor microenvironment and host immune response following treatment may affect prognosis. Lymphocytes play an important role in regulating the host immune and anti-tumor responses (4). Tumor infiltrating lymphocytes (TILs) and peripheral blood lymphocytes (PBLs) have shown promise as predictive biomarkers for their association with improved clinical outcomes for various malignancies (5–8).

Favorable outcomes following neo-adjuvant chemoradiotherapy (neo-CRT) have led to the recognition of neo-CRT alongside definitive chemoradiotherapy (CRT) as a standard treatment option for locally advanced ESCC (9). Although historically viewed as immunosuppressive, CRT has been known to activate immune system through multiple mechanisms including initiating immunogenic cell death (ICD) and production and release of inflammatory factors into tumor microenvironment, leading to improved tumor antigen expression and presentation as well as infiltration by various immune cells (10–12). Peng et al. found that neo-adjuvant chemotherapy (NACT) was associated with increased CD8^+^ TIL levels in post-treatment tumors (13). Teng and colleagues found increased CD4^+^ and CD8^+^ but stable Foxp3^+^ TIL densities in tumors resected from patients who underwent CRT (6). Similarly, CD8^+^ TIL densities rise dramatically following CRT in patients with rectal cancer (5). However, alterations to specific PBL subsets during CRT remains unstudied, so little is known about their prognostic value in patients with ESCC.

The present study aimed to evaluate changes in PBL subpopulations among patients with ESCC undergoing CRT. We measured the proportion of each PBL subtype in peripheral blood specimens before treatment and during treatment using flow cytometry. Furthermore, we evaluated the potential value of PBLs for predicting clinical outcomes in patients with ESCC.

## Materials and Methods

### Study Population

This pilot study enrolled patients with ESCC who received neo-adjuvant chemoradiotherapy (weekly cisplatin and docetaxel chemotherapy for four cycles combined with radiotherapy with a dose of 40 Gy in 20 fractions) followed by radical surgery 4–6 weeks later or definitive chemoradiotherapy (weekly cisplatin and docetaxel chemotherapy for four cycles combined with radiotherapy with a dose of 60 Gy in 30 fractions) between 2015 and 2017. Informed consent was obtained from each patient to undergo blood sample collection before treatment and at the time when the total radiation dose of 40 Gy was reached.

All patients met several inclusion criteria: (i) diagnosis of ESCC based on pathologic evaluation; (ii) age older than 18 years; (iii) Karnofsky performance status of 70 or higher, (iv) receipt of radiation therapy (RT) using a conventional fraction; (v) availability of baseline clinical, laboratory, and follow-up data; (vi) no concomitant receipt of immunosuppressants or antitumor therapy and no prior malignant tumor removal; and (vii) survival time of at least 3 months following CRT. Patients were excluded if they had a history of other malignancy, had not completed CRT, or had evidence of distant metastasis. A total of 64 patients with ESCC were included in the study as of April 2017. All patients were staged according to the 8th edition of the American Joint Committee on Cancer (AJCC) Cancer Staging Manual: Esophagus and Esophago-gastric Junction. The study was approved by the medical ethics committee of the institute.

### Peripheral Blood Lymphocyte Phenotype Detection

Blood samples were obtained by venous puncture before treatment and at the time when the total radiation dose of 40 Gy was reached. Four-color flow cytometric analysis of peripheral lymphocytes was performed. For each patient sample, two tubes were labeled with the sample identification number and A or B. Each tube was aliquoted with 50 μL of mixed, anticoagulated whole blood. Next, a pipette was used to add 20 μL of BD Multitest CD3 FITC/CD8 PE/CD45 PerCP/CD4 APC reagent (catalog no 340503, BD Biosciences, San Jose, CA, USA) into the bottom of each tube labeled A. Then, 20 μL of BD Multitest CD3 FITC/CD16+CD56 PE/CD45 PerCP/CD19 APC reagent (catalog no 340503, BD Biosciences, San Jose, CA, USA) was added into B tubes using a pipette. All samples were mixed gently using a vortex, then incubated for 15 min in the dark at room temperature. Following incubation, 450 μL of lysing solution was added to each tube. After 15 min at room temperature protected from light, cells were washed twice with staining buffer to remove unbound antibodies. Afterward, the samples were immediately analyzed by flow cytometry.

CD45 recognizes human leucocyte antigens in peripheral blood. T cells were defined by CD3 expression and B cells by CD19 expression. T-lymphocyte subsets were identified by the presence of CD4 and CD8. CD56 and CD16 together facilitate identification of the natural killer (NK) lymphocyte population.

### Follow-Up and Statistical Analysis

After treatment completion, contrast-enhanced computed tomography (CT) scans of the chest were obtained within 3 months and then every 6 months. Overall survival (OS) was calculated from initiation of CRT to the date of death from any cause or the date of the last follow-up. Patient characteristics were evaluated using descriptive statistics. Weight loss was defined as >5% unintentional weight loss during the 3 months before disease diagnosis. The lymphocyte counts before treatment and during treatment were compared using paired *t*-tests. CD4^+^ or CD8^+^T cell proportion was defined as the percentage of CD4^+^ or CD8^+^T cells in the CD45^+^ lymphocytes. Changes in CD4^+^ T cell proportion before and after CRT was defined as CD4^+^ T cell ratio = post-treatment CD4^+^ T cell proportion**/**pretreatment CD4^+^ T cell proportion, and changes in CD8^+^ T cell proportion before and after CRT was defined as CD8^+^ T cell ratio = post-treatment CD8^+^ T cell proportion**/**pretreatment CD8^+^ T cell proportion. The log-rank test was used to compare Kaplan-Meier survival curves. Multivariate analyses were performed using Cox proportional hazards regression. All statistical analyses were performed using SPSS software version 20.0 (SPSS Inc., Chicago, IL) and Graph Pad Prism 5 (Graph Pad Software Inc., San Diego, CA, USA). A *P* < 0.05 was considered statistically significant for two-sided tests.

## Results

### Baseline Patient Characteristics

A total of 64 patients met study inclusion criteria and were fully evaluated. Blood specimens were obtained pre-treatment and during treatment for each patient. Of the 64 patients, 56 (87.5%) were men, and 19 (29.7%) had never smoked. The median patient age was 65 years (range, 47–82 years). Baseline patient characteristics are detailed in Table 1.

**Table 1:** Clinical characteristics of 64 patients with esophageal squamous cell carcinoma.

**Characteristic**	***N* (%) or median range**
**Sex**	
Male	56 (87.5)
Female	8 (12.5)
**Age (years)**	65 (47–82)
**KPS**	
>80	34 (53.1)
≤ 80	30 (46.9)
**Smoke**	
Ever	45 (70.3)
Never	19 (29.7)
**Stage**	
II	5 (7.8)
III	45 (70.3)
IVa	14 (21.9)
**Tumor location**	
Upper	15 (23.4)
Middle	41 (64.1)
Lower	8 (12.5)
**Weight loss**	
Yes	24 (37.5)
No	40 (62.5)
**Treatment**	
Radical CRT	54 (84.4)
Neo-CRT + surgery	10 (15.6)

In present study, 61 patients completed radiotherapy, and 63 completed chemotherapy. Treatment-related toxicities during CRT included nausea (82.8%), leucopenia (62.5 %), and esophagitis (81.2%). The most common (≥5%) grade 3 or 4 toxicity included leucopenia (7.8%), esophagitis (23.4%). No treatment-related Grade 5 toxicity occurred during CRT. Most patients recovered with supportive care. Four patients in this study discontinued treatment as a result of severe hematologic toxicity (*n* = 1) and esophagitis (*n* = 3).

### Lymphocyte Changes in Peripheral Blood After Chemoradiotherapy

**Figure 1:**
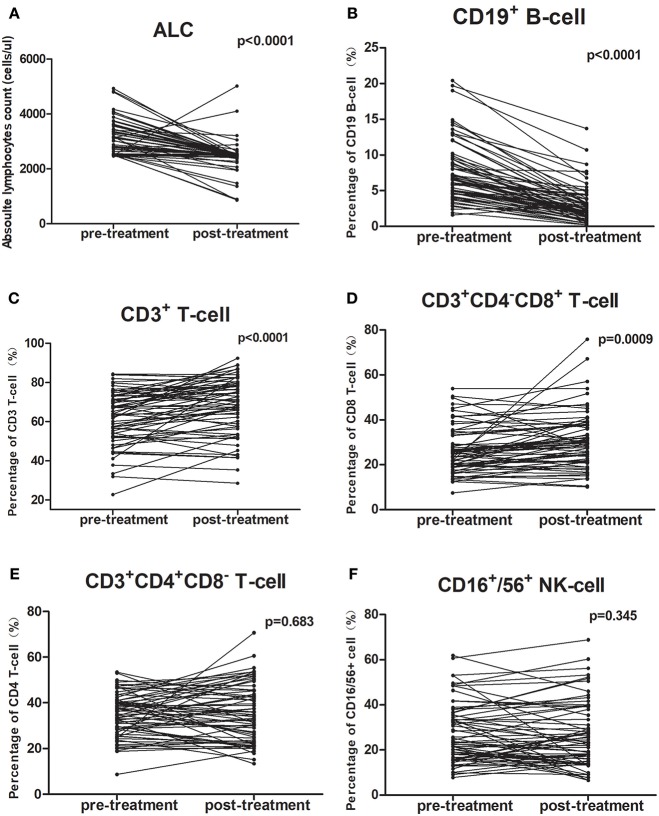
Chemoradiotherapy-induced alterations of circulating lymphocyte subpopulations for all cases. The proportion of each lymphocyte subpopulation before and during treatment were compared using paired *t*-test. **(A,B)** The mean absolute lymphocyte counts and proportion of CD19^+^ B cells were lower after CRT; **(C,D)** The proportions of CD3^+^ T-cells and CD3^+^CD4^−^CD8^+^ T-cells were higher after CRT; **(E,F)** The percentage of CD3^+^CD4^+^CD8^−^ T-cells and CD16^+^/56^+^ NK cells did not change after CRT.

All patients completed treatment. The changes in PBL subset proportions and absolute numbers in patients with ESCC are presented in Figure 1, Tables 2, 3. As shown, the proportion of CD19^+^ B cells decreased the most markedly following CRT, from 7.5 to 2.9% (*P* < 0.001). In contrast, proportions rose after treatment for CD8^+^ cells (26.1–30.6%; *P* = 0.001) and CD3^+^ cells (62.4–68.3%; *P* < 0.001). However, there were no changes in the proportions of CD4^+^ cells (34.8–35.4%; *P* = 0.683) or NK cells (26.7–27.7%; *P* = 0.345).

**Table 2:** Chemoradiotherapy-induced alterations of circulating lymphocyte subpopulation proportion for all cases.

**Variables**	**Pre-treatment**		**Post-treatment**		***P*-value**
***N* = 64**	**Mean**	**SD**	**Mean**	**SD**	**(paired *t*-test)**
CD19^+^ B cells (%)	7.5	4.2	2.9	2.8	< 0.001
CD3^+^ T cells (%)	62.4	13.5	68.3	14.6	< 0.001
CD3^+^CD4^−^CD8^+^ T cells (%)	26.1	10.3	30.6	12.6	0.001
CD3^+^CD4^+^CD8- T cells (%)	34.8	9.4	35.4	12.7	0.683
CD16^+^/CD56^+^ NK cells (%)	26.7	13.2	27.7	14.6	0.345

*SD, standard deviation*.

**Table 3:** Chemoradiotherapy-induced alterations of circulating lymphocyte subpopulation counts for all cases.

**Variables**	**Pre-treatment**		**Post-treatment**		***P*-value**
***N* = 64**	**Mean**	**SD**	**Mean**	**SD**	**(paired *t*-test)**
CD45^+^ lymphocyte counts	3081.0	636.9	2445.5	569.0	< 0.001
CD19^+^ B cells	240.3	152.6	75.1	79.6	< 0.001
CD3^+^ T cells	1926.0	584.9	1680.3	597.0	0.004
CD3^+^CD4^−^CD8^+^ T cells	806.6	361.4	775.2	495.6	0.623
CD3^+^CD4^+^CD8- T cells	1073.9	370.0	850.2	325.9	< 0.001
CD16^+^/CD56^+^ NK cells	845.5	419.1	632.5	359.1	< 0.001

*SD, standard deviation*.

The mean absolute lymphocyte counts decreased from 3,081 before CRT to 2,445 after CRT (*P* < 0.001). Moreover, the absolute counts of CD19^+^ B cells, CD16^+^/CD56^+^ NK cells, CD3^+^ T cells, and CD4^+^ T cells also decreased after CRT (*P* < 0.05, respectively). However, there was no significant changes in the absolute counts of CD8^+^ T cells (*P* = 0.623).

### Prognostic Significance of Chemotherapy-Induced Changes in CD4^+^ and CD8^+^ T-Cell Ratios in Patients With Esophageal Squamous Cell Carcinoma

The median follow-up period was 11.8 months (range, 4.0–20.2 months). At last follow-up, 12 patients (18.7%) had died with disease progression, 14 (21.9%) were alive with disease progression, and 38 (59.4%) were alive without progression.

**Figure 2:**
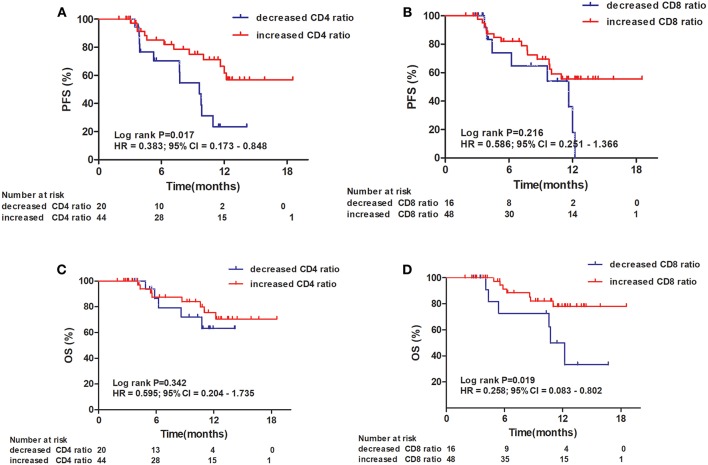
Progression-free survival (PFS) and overall survival (OS) of patients with esophageal squamous cell carcinoma. Progression-free survival curves for patients by CD4^+^ T-cell ratio **(A)** and CD8^+^ T-cell ratio **(B)**. Overall survival curves for patients by CD4^+^ T-cell ratio **(C)** and CD8^+^ T-cell ratio **(D)**.

An increased CD4^+^ T-cell ratio after CRT correlated closely with superior PFS (Figure 2A; hazard ratio [HR] = 0.383; 95% CI = 0.173–0.848, *P* = 0.017), while CD8^+^ T-cell ratio was not associated with PFS (Figure 2B, *P* = 0.216). In univariate analysis, TNM stage, tumor location, and increased CD4^+^ T-cell ratio were associated with PFS. However, only increased CD4^+^ T-cell ratio (*P* = 0.042) and TNM stage (*P* = 0.029) were independent predictors of PFS on multivariate analysis (Table 4). Similarly, an increased CD8^+^ T-cell ratio after CRT was associated with improved OS (Figure 2D; HR = 0.258; 95% CI = 0.083–0.802, *P* = 0.019), while CD4^+^ T-cell ratio showed no predictive significance (Figure 2C, *P* = 0.342). More importantly, multivariate analysis showed that increased CD8^+^ T-cell ratio (*P* = 0.040) was the only independent predictor of OS (Table 5).

**Table 4:** Univariate and multivariate analysis of progression-free survival.

	**Univariate analysis**			**Multivariate analysis**		
**Characteristics**	**HR**	**95%CI**	***P***	**HR**	**95%CI**	***P***
**Gender**						
Male vs. Female	0.224	0.030–1.656	0.143			
**Age**						
≥65 vs. < 65	0.558	0.221–1.414	0.219			
**Smoke**						
Never vs. Ever	1.259	0.525–3.021	0.606			
**Weight loss**						
Yes vs. No	0.998	0.448–2.226	0.997			
**Treatment**						
CRT vs. CRT+surgery	0.532	0.159–1.782	0.306			
**KPS score**						
≤ 80 vs. >80	0.667	0.275-1.614	0.369			
**TNM**						
II–III vs. IV	2.726	1.360–5.464	0.005	2.319	1.104–4.793	0.029
**Tumor location**						
Upper		Ref.				
Middle	5.079	1.168–22.083	0.030			
Lower	6.583	1.275–33.992	0.024			
**CD19**^**+**^ **B-cell ratio**						
Low vs. High	0.344	0.103–1.153	0.084			
**CD4**^**+**^ **T-cell ratio**						
Low vs. High	0.383	0.173–0.848	0.017	0.425	0.182–0.975	0.042
**CD8**^**+**^ **T-cell ratio**						
Low vs. High	0.586	0.251–1.366	0.216			
**CD16**^**+**^**/56**^**+**^ **cell ratio**						
Low vs. High	0.692	0.309–1.550	0.371			

*KPS, Karnofsky performance status; HR, hazards ratio; CI, confidence interval; Ref, reference*.

**Table 5:** Univariate and multivariate analysis of overall survival.

	**Univariate analysis**			**Multivariate analysis**		
**Characteristics**	**HR**	**95%CI**	***P***	**HR**	**95%CI**	***P***
**Gender**						
Male vs. Female	1.013	0.226–4.532	0.987			
**Age**						
< 65 vs. ≥65	1.721	0.590–5.016	0.320			
**Smoke**						
Never vs. Ever	1.155	0.362–3.692	0.808			
**Weight loss**						
Yes vs. No	0.558	0.151–2.065	0.383			
**Treatment**						
CRT vs. CRT+surgery	0.034	0.000–9.020	0.235			
**KPS score**						
≤ 80 vs. >80	0.813	0.357–1.851	0.623			
**TNM**						
II–III vs. IV	2.873	0.995–8.669	0.045			
**Tumor location**						
Upper		Ref.				
Middle	4.762	0.601–37.718	0.139			
Lower	10.485	1.169–94.003	0.036			
**CD19**^**+**^ **B-cell ratio**						
Low vs. High	0.484	0.108–2.169	0.343			
**CD4**^**+**^ **T-cell ratio**						
Low vs. High	0.595	0.204–1.735	0.342			
**CD8**^**+**^ **T-cell ratio**						
Low vs. High	0.258	0.083–0.802	0.019	0.317	0.105–0.951	0.040
**CD16**^**+**^**/56**^**+**^ **cell ratio**						
Low vs. High	0.464	0.162–1.327	0.152			

*KPS, Karnofsky performance status; HR, hazards ratio; CI, confidence interval; Ref, reference*.

**Figure 3:**
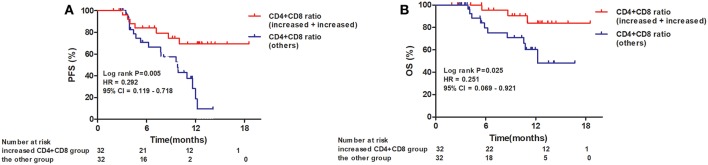
Kaplan–Meier survival curves for overall survival and progression-free survival based on both increased CD4^+^ T-cell and CD8^+^ T-cell ratios compared to all others. **(A)** Progression-free survival curves for patients by combined CD4^+^ and CD8^+^ T-cell ratios; **(B)** Overall survival curves for patients by combined CD4^+^ and CD8^+^ T-cell ratios.

When the 64 patients with ESCCs were separated into two groups, the 32 patients with both increased CD4^+^ and CD8^+^ ratios had a superior PFS and OS, compared to the 32 patients with an increased CD4^+^ ratio only or CD8^+^ ratio only or neither (1-year PFS rate 63 vs. 25%, 1-year OS rate 80 vs. 62%, *P* = 0.005 and 0.025, respectively). Representative Kaplan–Meier survival curves based on these factors are shown in Figures 3A,B.

## Discussion

Neoadjuvant CRT followed by surgery has been considered the standard treatment for locally advanced esophageal cancers, however, the optimal chemotherapy regimen remains controversial (9, 14, 15). Docetaxel is well-known to be a potent radiosensitizer. A phase II trial reported that concurrent CRT with weekly docetaxel and cisplatin was well-tolerated and resulted in favorable activity in terms of both tumor and symptom control (16). Moreover, Xi et al. suggested that, compared to traditional fluorouracil and cisplatin regimen, concurrent chemoradiotherapy with docetaxel and cisplatin in squamous cell esophageal carcinoma was associated with a satisfactory outcome and manageable toxicity (17). Collectively, these findings indicated that the combination of docetaxel and cisplatin for concurrent CRT in esophageal cancer is promising. Growing evidence suggests that CRT plays an important therapeutic role for patients with ESCC, however, factors predicting CRT efficacy, which would facilitate optimal disease management for patients with ESCC, have yet to be fully elucidated. Recent research supports the role of CRT in activating the tumor immune microenvironment to affect tumor response and prognosis (12, 18). Therefore, we hypothesized that circulating lymphocytes, crucial components of human antitumor immunity, may affect response to CRT. This prospective study is the first to measure CRT-induced changes in lymphocyte subpopulations and their prognostic value in ESCC. Our study showed that CRT can activate the immune system and that CD4^+^ and CD8^+^ T-cells are positively associated with superior PFS and prolonged survival.

Lymphocytes account for ~30% of the normal human white blood cell population and play an important role in antitumor immunity (4). PBLs are well-known to be extremely sensitive to radiation. Even low-dose total body radiation can decrease circulating lymphocyte counts, which in turn may compromise antitumor immune responses during CRT (19, 20). Previous reports involving non-small cell lung cancer and unresectable hepatocellular cancer, demonstrated a significant correlation between lymphocyte nadirs and radical RT (8, 21). However, the radiosensitivity of different lymphocyte subsets seems to be inconsistent. Stereotactic body radiotherapy for early stage non-small cell lung cancer suppresses all lymphocyte subset counts for 1–4 weeks following treatment completion (22). Our results showed that absolute PBL counts declined after CRT, and that the drop was most pronounced in the B-cell subgroup. This finding was consistent with previous studies that show B-lymphocytes to be the most radiosensitive subpopulation in non-hematologic malignancies. This sensitivity may be caused by the Ku86 protein variant that has a decreased ability to recruit the catalytic component of the DNA-protein kinase complex, which is crucial to DNA double-strand break repair (23). In addition to its immunosuppressive effects, CRT has been known to be immune stimulatory to enhance anti-tumor immune response (10–12). Previous studies that show a positive correlation between CRT and proliferative CD8^+^ TIL activity (24). Our study also found that tumor-specific CD8^+^ cytotoxic T lymphocytes (CTLs) levels increased after CRT.

Accumulated clinical studies demonstrated a closely association with high lymphocytes and better patient outcomes in several types of human cancer (8, 21). Previous research on T-cells has long been focused on tumor-specific CD8^+^ CTLs because of their potent killing activity, while CD4^+^ T-cells have been studied primarily for their role as helpers to CD8^+^ CTLs. Recent studies indicated that CD4^+^ T cells were not a pure cell lineage with a single function, but a diverse cell population with complex functions (25, 26). Moreover, CD4^+^ T cells may serve not only as helper cells, but also as potent effector cells or partners with macrophages and eosinophils to clear a wide variety of tumors (27, 28). Our results showed that CRT-induced increases in peripheral CD4^+^ and CD8^+^ T cell levels are a surrogate marker for immunostimulation and are associated with superior survival in patients with ESCC. Furthermore, our study revealed that the prognosis of patients with both increased CD4^+^ and CD8^+^ T-cell ratios was remarkably better than that of patients with an increased CD4^+^ ratio only or CD8^+^ ratio only or neither. The finding was consistent with the well-established cooperative role of CD4^+^ and CD8^+^ T-cells in tumor eradication (29). Therefore, these results indicated the combination of CD4^+^ and CD8^+^ T-cell could be a promising and valuable prognostic marker of anti-tumor immune response after CRT. It may provide the rationale for multipronged approaches to combine CRT with immunotherapy in patients with ESCC, with the goal of enhancing immune response more efficiently to improve survival.

In addition, CRT-induced CD19^+^ B-cell decreases in our study weakened humoral immunity and presumably resulted in worse outcomes for patients with ESCC (but this difference did not reach statistical significance). In patients with cancer, CD19^+^ B lymphocytes play an important role in humoral immunity through their specific binding to B-cell activating factor (BAFF) and production of antibodies for tumor-associated antigen. Moreover, B cells can also process and present antigens to induce T cell immune responses and interact with macrophages and the complement system to kill tumor cells (30–32). Notably, natural killer (NK) cells are a small subset of cytotoxic lymphocytes which contribute to innate and adaptive immunity through cytolysis and release of chemokines and large amounts of T-helper cytokines such as IFN-γ (33). Contrary to previous reports involving non-small cell lung cancer, gastric cancer, and colorectal cancer (34, 35), NK cells were not a marker of improved prognosis in this study. Future research is warranted, therefore, to elucidate the relationship between B-cells and NK cells and clinical outcomes.

Some limitations deserve mention. Firstly, our sample size is relatively small, and patients had a short duration of follow-up. Secondly, additional blood collection time points testing of antigen specific CD4^+^ and CD8^+^ T cells may allow for more precise characterization of lymphocyte alterations. Further prospective studies are warranted to validate our findings.

## Conclusion

In summary, this study was conducted to evaluate the effects of CRT on PBL subpopulations and evaluate possible clinical implications for patients with esophageal cancer. Moreover, multivariate analysis revealed that CRT-induced alterations of CD4^+^ and CD8^+^ T-cells are valuable and promising predictors of survival. This finding suggests that CRT might activate the immune response, thereby affecting treatment response and prognosis. Thus, we propose that immune therapy during CRT may promote anti-tumor immune response to confer a survival benefit to patients with esophageal squamous cell carcinoma.

## Ethics Statement

This study was carried out in accordance with the recommendations of national ethical guidelines. The protocol was approved by the Medical Ethics Committee of Tianjin Medical University Cancer Institute and Hospital. All subjects gave written informed consent in accordance with the Declaration of Helsinki.

## Author Contributions

XC and WZ have designed the paper, performed experiments, and wrote the main manuscript. DQ, YG, CY, and XR have been part of every step in this patients' complicated diagnostic and therapeutic course and gave valuable interpretation of data. YL performed flow cytometric analysis of blood samples. YW, HZ, and PE collected clinical data and conducted follow-up. QP and PW designed and directed the overall project. All the coauthors revised paper critically and gave final approval of this version for publishing. They have ensured that all aspects of the work are accurate and have been appropriately investigated and resolved.

### Conflict of Interest Statement

The authors declare that the research was conducted in the absence of any commercial or financial relationships that could be construed as a potential conflict of interest.
